# Triclosan Adsorption on Chitosan: Computational Study of Molecular Interactions and Potential for Environmental Remediation

**DOI:** 10.3390/polym17040487

**Published:** 2025-02-13

**Authors:** Cleidiane Cardoso Teixeira, Anna Karla dos Santos Pereira, Grasiele Soares Cavallini, Douglas Henrique Pereira

**Affiliations:** 1 Postgraduate Program in Chemistry, Department of Chemistry, Federal University of Tocantins, Campus Gurupi-Badejós, P.O. Box 66, Gurupi 77402-970, TO, Brazil; cleidiane.teixeira.cardoso@gmail.com (C.C.T.); anna_karla@mail.uft.edu.br (A.K.d.S.P.); grasiele@mail.uft.edu.br (G.S.C.); 2 Department of Chemistry, Technological Institute of Aviation, Praça Marechal Eduardo Gomes, 50, Vila das Acácias, São José dos Campos 12228-900, SP, Brazil

**Keywords:** triclosan, adsorption, chitosan, emerging contaminant

## Abstract

The compound triclosan (TCS) is widely found in personal hygiene products, and when present in effluents, it can cause problems to human health, such as endocrine disruption, intestinal problems, and liver tumors. A sustainable alternative for the removal of TCS is the use of adsorbent biopolymers, which are low-cost, renewable, and biodegradable. One of the most widely used biopolymers is chitosan (CHT), which has excellent adsorptive properties due to its functional groups. In this context, the present work evaluated, through computational simulations, the interaction of the TCS molecule with CHT. The frontier molecular orbitals and the molecular electrostatic potential show that different forms of interactions can occur, and thus, five complexes were shown to be stable after the optimization of the interactions. The bond lengths of the interactions ranged from 1.839 Å to 3.606 Å and were formed mainly by hydrogen bonds and H^...^Cl interactions. The binding energy (∆E_Bind_) allowed us to infer that adsorption occurred, ∆E_Bind_ < 0, and the values ranged from −4.14 kcal mol^−1^ to −17.74 kcal mol^−1^. The thermodynamic properties demonstrated that the process was exothermic and that two complexes were spontaneous: TCS^...^CHT^iii^ with ΔG= −3.14 kcal mol^−1^ and TCS^...^CHT^iv^ with ΔG= −2.82 kcal mol^−1^. The topological parameters revealed that almost all interactions between TCS and CHT were electrostatic, and the non-covalent interaction analysis confirmed the presence of van der Waals interaction between the complexes. Thus, it can be confirmed that this study showed the efficient use of chitosan for the treatment of effluents containing the emerging contaminant triclosan.

## 1. Introduction

2,4,4′-trichloro-2′-hydroxydiphenyl ether (C_12_H_7_Cl_3_O_2_), known as triclosan (TCS), is an antibacterial substance that can be found in personal hygiene products such as deodorants, toothpastes, soaps, and also cosmetics. Its frequent use has caused great concern, as it can cause problems to human health and the environment [1].

Triclosan contamination in aquatic environments can occur through the release of wastewater from wastewater treatment plants (WWTPs) into water bodies and into the soil through irrigation with contaminated wastewater and surface water [2]. Due to its lipophilic and bioaccumulative characteristics, TCS enters the food chain through plant absorption and can reach animals and, consequently, affect human health [3,4].

TCS poisoning can cause several problems to human health, such as endocrine disruption, intestinal problems, and liver tumors [5]. In aquatic species, TCS exposure can cause mutagenicity, carcinogenicity, and immunogenicity [6]. The presence of TCS in the soil affects the structure and function of microorganisms and inhibits the biodegradation of other substances [7]. In addition to direct contamination, TCS can be transformed through ultraviolet radiation into dioxins such as 2,8-dichlorodibenzene-p-dioxins, which are highly toxic contaminants [8].

TCS is found in low concentrations in the environment; however, the acceptable daily intake of TCS is 0.17 nmol/kg/day [2]. Furthermore, evidence shows that conventional WWTP treatment processes are not sufficient to completely remove this contaminant in the contamination of surface water by treated domestic sewage, which is why the use of advanced treatments is necessary [9]. In this context, the adsorption process stands out, which has the advantage of using different materials, such as activated carbon, coffee beans, pineapple stems, olive pits, chitosan, and minerals, among others [10,11]. Adsorptive materials such as biopolymers are sustainable alternatives, have a low cost, and stand out in the removal of different contaminants [12]. Chitosan (CHT), in particular, has high applicability in several areas, and it is the second most abundant renewable and biodegradable biopolymer, being obtained through the deacetylation of chitin [13].

Chitosan is the main derivative of chitin; it is formed by a heterogeneous structure composed of 2-acetamido-2-deoxy-β-d-glucopyranose bonded to 1–4 and 2-amino-2-deoxy-β-d-glucopyranose and differs from chitin by the presence of an amino group in the chain, making CHT soluble in weak acids [14,15]. CHT has essential functional groups in its structure, which include primary amino groups and primary and secondary hydroxyl groups, which are very reactive. In addition, there are also acetamide groups and glycosidic bonds, and these groups have opportunities for modification, which allows the creation of polymers and composites with different properties [16,17].

CHT has physicochemical and biological properties that make it an advantageous polymer. In terms of physicochemical properties, it is a cationic biopolymer with many functional groups for chemical and crosslinking reactions. It has chelating, complexing, and adsorption properties, and can also form intra- and intermolecular hydrogen bonds. In terms of biological properties, the polymer is non-toxic and has antacid, antiulcer, and antitumor properties, among others [15,16,17,18].

In conjunction with experimental data, computational simulations have proven to be interesting alternatives in the study of adsorptive processes, as they offer an understanding of the process at the atomic/molecular level, allow the evaluation of different adsorbent matrices and adsorbates in relation to their toxicity and costs and also allow the prediction of whether the process is thermodynamically and kinetically viable [19]. Therefore, the present work aimed to theoretically evaluate the interaction of the emerging contaminant TCS with the chitosan matrix, evaluating how the TCS-CHT interaction occurs and determining its structural, energetic, and topological properties. With the results of the present work, the molecular aspects of the TCS-CHT adsorption process will be evaluated in detail, providing new insights with implications for the use of adsorbent biopolymers, improvements to the adsorbent matrix, and use in environmental remediation, thus reducing financial costs and research time.

## 2. Materials and Methods

Density functional theory (DFT) [20] was used to calculate the interaction of TCS with CHT, and the calculations were performed with the Gaussian 16, Revision C.02, program [21]. For all the work, the ω-B97XD method [22,23] with the 6-31 + G(d,p) basis set [24,25] was used. The ω-B97XD method includes empirical atom–atom dispersion corrections, which is an important effect for studying interactions. The GaussView program [26] was used in the design of the structures and also to generate the frontier molecular orbitals (FMOs) and the molecular electrostatic potentials (MEPs). The isolated TCS and CHT molecules and their interactions were optimized, and no imaginary frequencies were found, evidencing that the structures were at the energy minimum. The effect of water as a solvent was simulated with the implicit solvent SMD (solvation model based on density) [27].

The HOMO (highest occupied molecular orbital) and LUMO (lowest unoccupied molecular orbital) values of the isolated molecules were calculated, and the energy gap value was determined by Equation (1).

∆E_gap_ = E_LUMO_ − E_HOMO_ (1)

To quantify the interactions between the adsorbent and adsorbate, the binding energy (ΔE_Bind_) was calculated via Equation (2). Negative values indicated that interactions are effective and positive values that they were not. Thermodynamic properties are also important in studies on adsorption processes, where the variations in enthalpy and Gibbs energy are used to evaluate whether the adsorption is endothermic or exothermic, and whether it is spontaneous or not, using Equations (3) and (4).

ΔE_Bind_ = E_CHT-TCS_ − (E_CHT_ + E_TCS_) (2)

Δ_r_H = H_CHT-TCS_ − (H_CHT_ + H_TCS_) (3)

Δ_r_G = G_CHT_-_TCS_ − (G_CHT_ + G_TCS_) (4)

where subscripts CHT-TCS, CHT, and TCS refer to the energy, enthalpy, or Gibbs free energy of the complex, the adsorbent, and the adsorbate, respectively. To correct the method error, the zero-point energy (ZPE) was added to the electronic energies (E_CHT-TCS_, E_CHT_, and E_TCS_), and no scale factor was used in the frequency calculations.

The topological data of the systems were described by quantum theory of atoms in molecules (QTAIM) at the critical bonding points of the interactions. The parameters calculated the QTAIM were ρ(r) (electron density), ∇^2^ρ(r) (Laplacian of the electron density), G(r) (kinetic energy), V(r) (potential electron energy density), and H(r) (total electron energy density). The AIMALL package was used for the analysis [28].

The isosurface function of the reduced density gradient (RDG) was used to calculate the non-covalent interaction index (NCI) using Equation (5).

(5) $$RDG=\frac{1}{{2({3\pi }^{2})}^{1/3}}\frac{\left|\nabla \rho (r)\right|}{{\rho (r)}^{4/3}}$$

The Multiwfn 3.8 software [29] was used to perform the NCI calculations, and the Visual Molecular Dynamics 1.9.4 (VMD) software [30] was used to visualize the results obtained.

## 3. Results

In adsorptive processes, variables such as temperature, pH, interferents, zeta potential, and others can affect the process. In quantum chemistry simulations, the system size, as well as the evaluation of conditionals, can significantly increase the computational cost, making the execution of simulations unfeasible. In this context, to study the adsorption process of the chitosan matrix with the emerging contaminant TCL, a CHT matrix composed of three monomeric units was used. Hydrogens were added to complete the valence of the matrix, and all simulations were carried out with neutral molecules.

### 3.1. Frontier Molecular Orbitals and Molecular Electrostatic Potential

**Figure 1:**
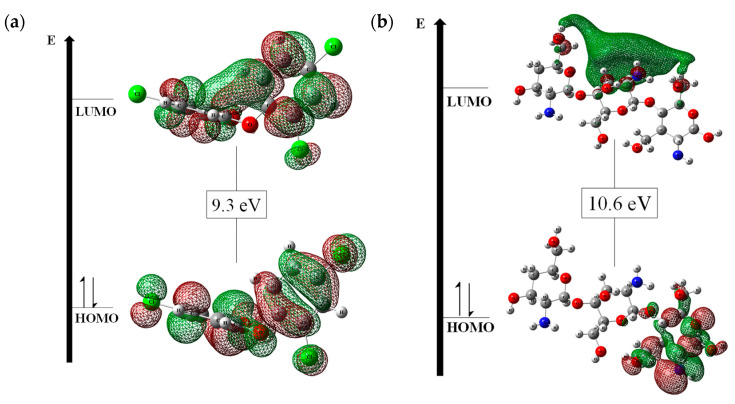
HOMO–LUMO frontier molecular orbitals for (**a**) triclosan (TCS) and (**b**) chitosan (CHT).

Frontier molecular orbitals, especially HOMO-LUMO, provide several insights into chemical compounds, allowing the evaluation of the electron probability density and assessing molecular stability and reactivity [31]. Thus, the FMOs for TCS and CHT were evaluated, and the results are shown in Figure 1. Analyzing the results in Figure 1a, it is possible to observe that TCS presents well-defined π orbitals (HOMO and LUMO) throughout the molecule.

Analyzing Figure 1b, it is possible to observe that the HOMO of the chitosan presents a π orbital in one of the rings of the structure, while the LUMO presents a high probability density of finding the electron above the central ring of the structure.

The practical inference that can be made from the results in Figure 1 of the FMOs is that the energy gap values found for TCS and CHT were 9.3 eV and 10.6 eV, respectively, indicating that these chemical species are very stable because a larger energy gap correlates with greater molecular stability. Additionally, the ∆Egap value for CHT suggests that the molecule is a good insulator and that there may be chemical interactions between the molecular orbitals of the molecules, which implies that the adsorption process could occur.

**Figure 2:**
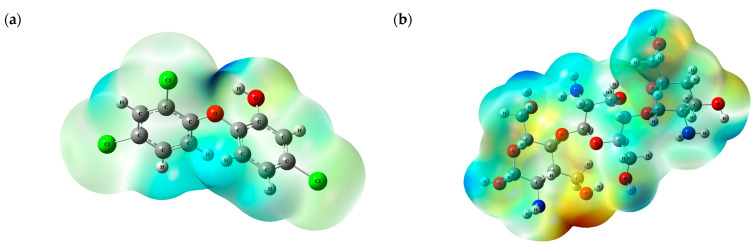
Molecular electrostatic potential for (**a**) triclosan and (**b**) chitosan.

Another important analysis is the molecular electrostatic potential, which provides information about chemical and molecular reactivity through the electron density of the molecule. The interpretation of the MEP is performed by identifying the colors, where the reddest region indicates a negative charge and, therefore, the presence of electrons; regions with a blue color indicate a low electron density, presenting a positive charge; and regions with a greenish color indicate neutrality [32]. Figure 2 shows the MEPs of the adsorbent–adsorbate.

Analyzing the MEPs, Figure 2, it is possible to observe that the region of the molecules where the blue color is concentrated is predominated by the hydrogen atom, that is, this region can interact with electronegative regions. The reddish region found in the molecules is due to the oxygen and chlorine atoms and also the nitrogen atom of the CHT, regions that can interact with positive parts.

With the results of the FMOs and MEPs, it is possible to infer that (i) there may be interactions between chitosan and triclosan; (ii) these interactions may occur through hydrogen bonds; and (iii) interactions may occur through the orbitals of the aromatic ring and the hydrogens of the TCS.

### 3.2. Structural Properties

**Figure 3:**
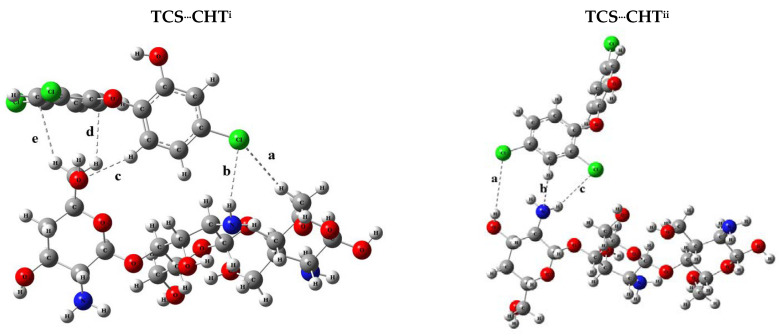

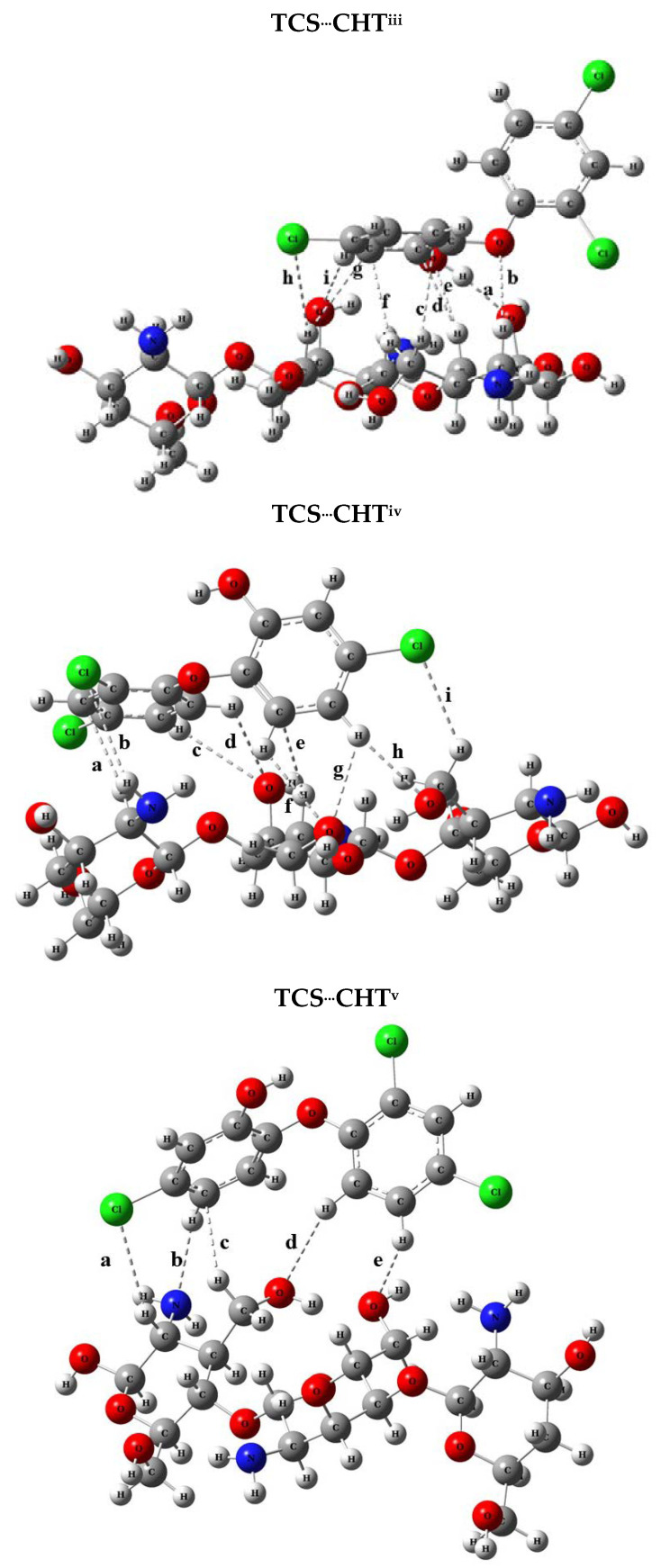
Structural configurations of the systems formed between triclosan and chitosan: TCS^...^CHT^i^, TCS^...^CHT^ii^, TCS^...^CHT^iii^, TCS^...^CHTi^v^, and TCS^...^CHT^v^.

With the results of the MEP and FMOs, it was possible to evaluate the different possibilities of TCS^...^CHT interactions that may occur. To evaluate all possibilities, the CHT matrix was positioned, and the TCS molecule was placed on the surface in five different ways to evaluate the main interaction possibilities. All TCS^...^CHT interactions were optimized without any geometry restrictions, letting the structures move to the most stable configurations. Thus, five stable complexes were formed, and the complexes were named TCS^...^CHT^i^, TCS^...^CHT^ii^, TCS^...^CHT^iii^, TCS^...^CHT^iv^, and TCS^...^CHT^v^ and are represented in Figure 3 with the molecular interactions highlighted.

Figure 3 shows the possible interactions that may occur between the adsorptive matrix and the contaminant, and the results of the interaction distances obtained for each system are shown in Table 1. In general, it is possible to analyze that the bond lengths varied from 1.839 Å to 3.606 Å. Analyzing each system separately, it is possible to observe that for the TCS^...^CT^i^ complex, five interactions were formed, in which the interaction with the shortest bond length was the O53-H89 hydrogen bond, interaction “c” with a value of 2.484 Å. The TCS^...^CT^ii^ complex presented only three interactions that occurred between H^...^Cl and between N^...^H.

For TCS^...^CHT^iii^, the interaction “a” that occurred between O58-H94 was the one that obtained the lowest value, with a distance of 1.839 Å, and this complex presented nine interactions. The TCS^...^CHT^iv^ complex also had nine interactions, in which the H^...^O hydrogen bonds presented the smallest bond length values. Finally, the TCS^...^CHT^v^ system presented five interactions, and the N^...^H interaction was the one that presented the smallest bond length of 2.447 Å.

With the results of the structural properties, bond lengths, and interactions, it is possible to assess that the -NH_2_ and -OH groups of the CHT stand out as being the interacting groups of the adsorptive matrix. There were also interactions between the chlorine atoms and the hydrogens in the CHT and also interactions between the hydrogens of TCL and the oxygens of the matrix. These data reveal that the predominant interactions were hydrogen bonds and H^...^Cl interactions. Another important point to highlight is that the contaminant in the aqueous solution can be in movement, and depending on the active site and orientation, it can interact and present different numbers of adsorbate–adsorbent interactions, as shown in Table 1.

### 3.3. Binding Energy, Enthalpy, and Gibbs Energy

When evaluating molecular interactions, it is important to analyze all the energetic and thermodynamic parameters to determine whether the adsorption process is viable. To verify the strength of the interactions, whether the process is exothermic or endothermic and spontaneous or not, the binding energies (ΔE_Bind_), the enthalpy, and the Gibbs energy variations were calculated, respectively, as shown in Table 2.

Analyzing the results found, it is possible to infer that the ΔE_Bind_ values for the analyzed systems show that all interactions were effective (ΔE_Bind_ < 0). The most significant interaction was that of the TCS^...^CHT^iv^ complex with a value of −17.74 kcal∙mol^−1^, and the interaction with the lowest intensity was that of the TCS^...^CHT^ii^ complex with a value of −4.14 kcal∙mol^−1^.

In the adsorption process, it is essential to analyze the thermodynamic parameters, which are given by the enthalpy and the Gibbs energy variations. Thus, it is possible to observe that the enthalpy variation of all complexes was exothermic, where ΔH < 0. For the Gibbs energy variation, only TCS^...^CHT^iii^ and TCS^...^CHT^iv^ presented ΔG values below 0, which indicates that the process was spontaneous. The complexes TCS^...^CHT^i^, TCS^...^CHT^ii^, and TCS^...^CHT^v^ had ΔG values greater than 0; therefore, these interactions were non-spontaneous.

### 3.4. Quantum Theory of Atoms in Molecules (QTAIM)

**Figure 4:**
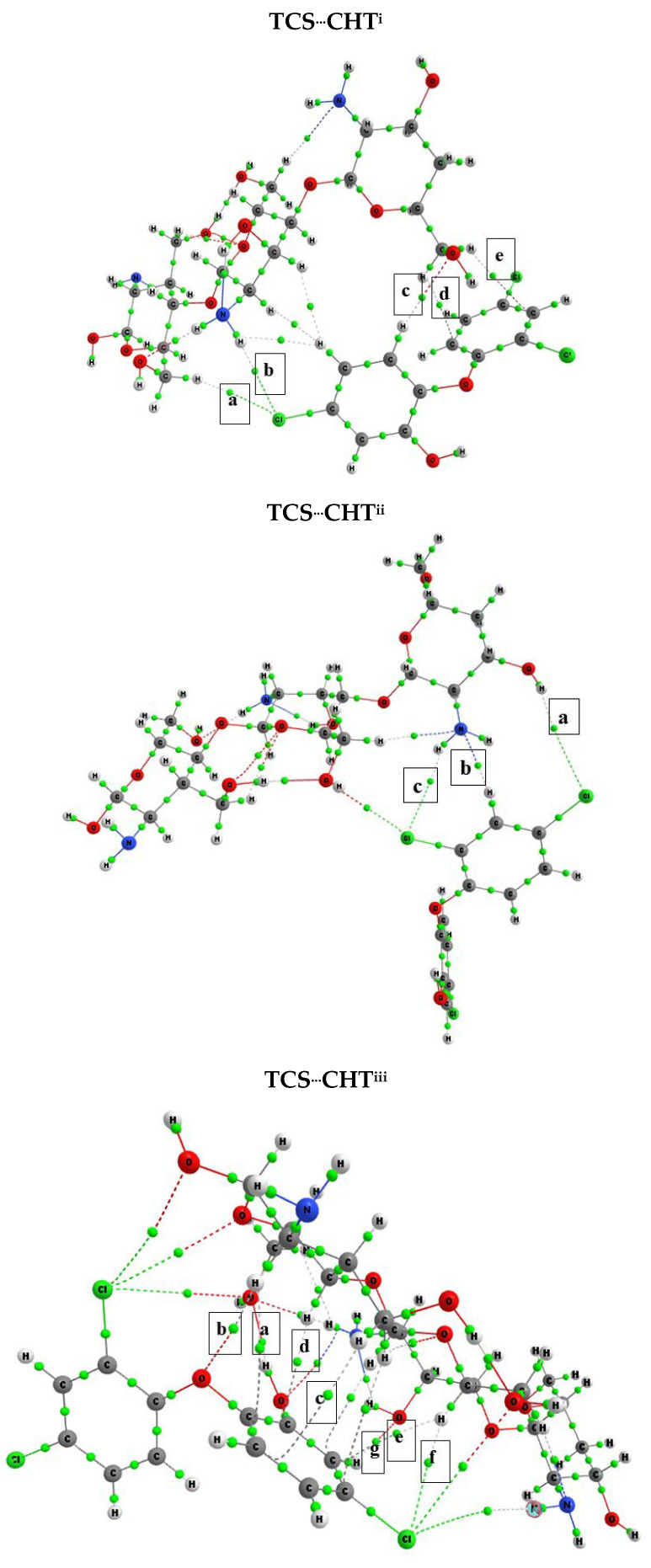

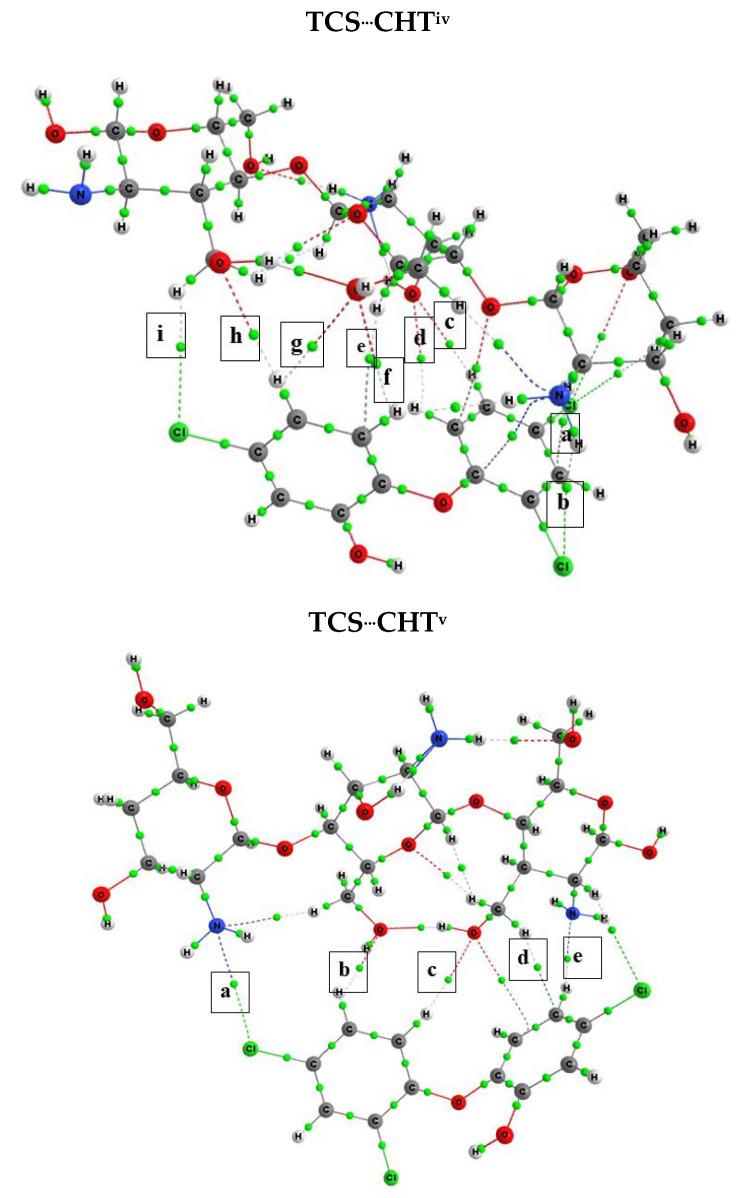
Molecular graphs generated by QTAIM highlighting the critical interaction points for the TCS^…^CHT^i-v^ complexes.

The quantum theory of atoms in molecules was developed by Bader and uses topological properties to describe the electronic structure of molecules [33,34]. The QTAIM allows the identification of the bond critical point (BCP), which helps determine whether there is an interaction or chemical bond. Its topological properties enable the evaluation of the interaction strength and its character, providing insights into the electronic level of the systems [33,34]. In this context, the QTAIM was used to evaluate the interactions, and the molecular graphs generated, along with the respective critical bonding points between TCS and CHT, are represented in Figure 4. The values of the topological parameters found are shown in Table 3.

In Figure 4, the molecular graphs generated by the QTAIM are represented, highlighting the effective interactions between the adsorbate and adsorbent. The other BCPs of the graphs were not considered as they did not adequately describe the interactions. The topological properties results in Table 3 indicate that all BCPs corresponded to a non-covalent interaction because ∇^2^ρ(r) > 0 and the electron density value (ρ(r)) was small (<0.1 au). Thus, by analyzing the results for the complexes, it is possible to infer that all the highlighted BCPs were molecular interactions.

Molecular interactions can also be classified using the parameters ∇^2^ρ(r) and H(r). When ∇^2^ρ(r) > 0 and H(r) < 0, the interaction is classified as partially covalent, and when ∇^2^ρ(r) > 0 and H(r) > 0, it is classified as electrostatic [33,34,35,36]. All interactions of the CHT^...^TCS complexes were electrostatic, except for two interactions: interaction “a” of the TCS^...^CHT^iii^ complex and interaction “b” of the TCS^...^CHT^v^ complex, which were partially covalent. These results indicate that the adsorption process is the result of almost all electrostatic interactions; therefore, it will have a reversible interaction.

From the QTAIM results, it is also possible to quantify the hydrogen bond energies (E_HB_) of the interaction at the critical bonding point using Equation (6) [33,34,35,36].

E_HB_ = 1/2 |V(r)|BCP (6)

For the TCS^...^CHT^i-v^ complexes, hydrogen bonds occurred between oxygens and hydrogens and between nitrogen and hydrogens, and the E_HB_ found for the interactions ranged from 0.7 kcal mol^−1^ (TCS^...^QT^iv^, interaction d) to 14.28 kcal mol^−1^ (TCS^...^QT^iii^, interaction “b”).

### 3.5. Non-Covalent Interactions (NCIs)

The QTAIM analyses showed that almost all interactions were electrostatic. In this context, another important analysis technique is non-covalent interactions (NCIs). NCI analyses are crucial because they refer to interactions between molecules that do not involve electron sharing. These interactions include van der Waals, hydrophobic interactions, hydrogen bonds, and dipole–dipole interactions, among others [37,38].

**Figure 5:**
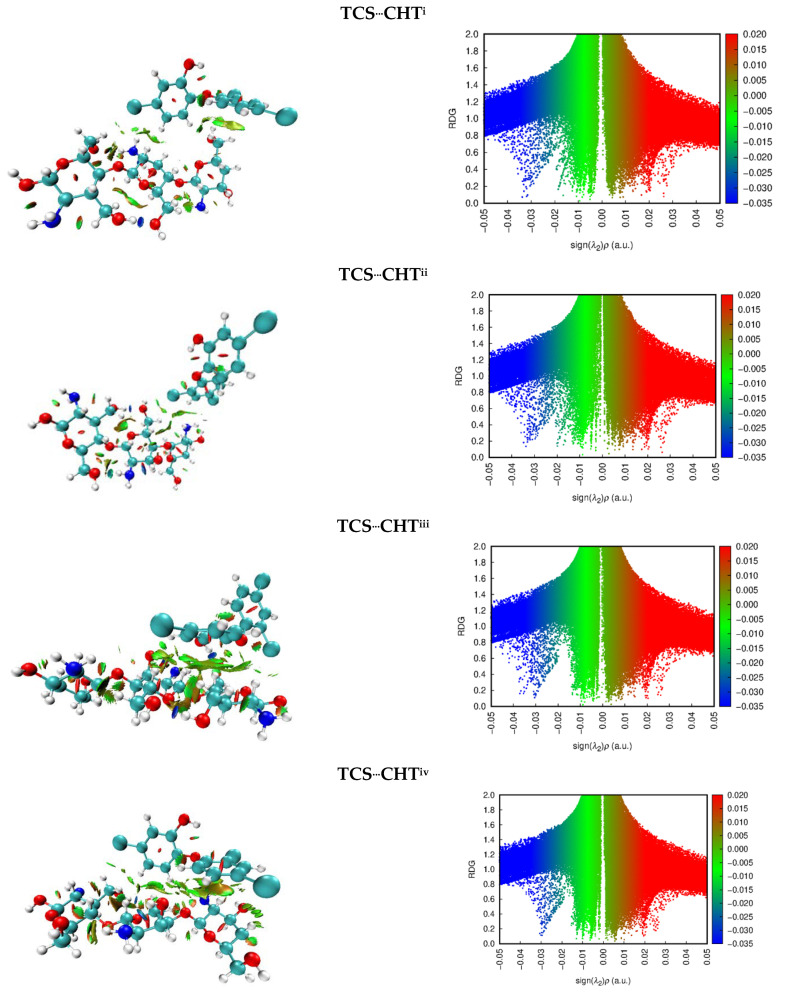

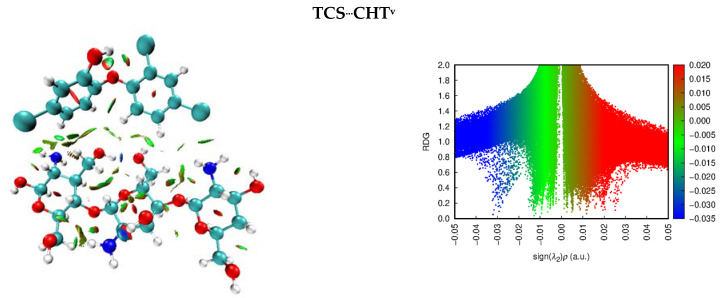
Three-dimensional non-covalent interaction plots (**left**) and two-dimensional plots (**right**) of the reduced density gradient versus the electron density multiplied by the sign of the second Hessian eigenvalue for TCS^...^CHT^i-v^ complexes.

NCIs are a tool used to make it possible to expand the results obtained by the QTAIM. Thus, all systems were evaluated by NCIs, and the results are represented in Figure 5. To analyze the interactions, two parameters were evaluated: the electron density of peaks (λ2) and the electron density (ρ(r)). These parameters provide information on the strength and type of interaction [37]. Interactions with values of sign(λ2)ρ << 0 indicate hydrogen bonding or an attractive interaction; interactions with values of sign(λ2)ρ close to or equal to 0 are described as van der Waals interactions; and interactions with values of sign(λ2)ρ >> 0 describe steric repulsion. Furthermore, it is possible to know the type of interaction through color coding: hydrogen bonds or attractive interactions are represented by the color blue, van der Waals are represented by the color green, and steric repulsions are represented by a reddish color, and, furthermore, the darker the color, the stronger the interaction [37,38].

Analyzing the results in Figure 5, it is possible to observe that the green color predominates among the triclosan–chitosan interactions, indicating van der Waals interactions. The RDG peaks vary approximately between 0.1 a.u. and 0.00 a.u., being within the expected range for intermediate interactions. All systems present red spheres in the center of the aromatic rings, which represents the steric effect. Only TCS^...^CHT^iii^ presents a bluish sphere between the adsorbate and adsorbents, indicating a strong hydrogen bond for this complex by NCIs.

## 4. Conclusions

The theoretical calculations based on density functional theory showed that triclosan can interact with the chitosan matrix. The analysis of the structural parameters showed that interactions occurred between the adsorbent and adsorbate, and the bond lengths of the interactions ranged from 1.839 Å to 3.606 Å. The energy analyses showed that the process was effective, the enthalpy of the reaction was exothermic, and two complexes presented Gibb’s energy values of < 0, therefore being spontaneous. The QTAIM topological analysis showed that almost all interactions were electrostatic, and the NCI analyses showed that the interactions were weak and of the van der Waals type for all complexes. Finally, considering the good results of the simulations, it is possible to infer that the CHT matrix is a good alternative for use in environmental remediation, especially for waters contaminated by triclosan, thus allowing its application in future experimental studies.

## Figures and Tables

**Table 1:** Structural analysis of interactions for complexes formed between triclosan and chitosan.

Complex	Interaction	Atoms	Distance (Å)
TCS^...^CHT^i^	a	H56-Cl95	3.143
	b	H38-Cl95	2.963
	c	O53-H89	2.484
	d	H51-C73	2.724
	e	H52-C76	2.975
TCS^...^CHT^ii^	a	H13-Cl81	3.606
	b	N14-H80	2.578
	c	H15-Cl82	3.262
TCS^...^CHT^iii^	a	O58-H94	1.839
	b	H63-O83	3.035
	c	H68-C86	3.143
	d	H44-C85	2.593
	e	H44-O93	2.681
	f	H69-C87	3.090
	g	H25-C87	2.930
	h	H25-Cl95	3.175
	i	O39-H91	2.541
TCS^...^CHT^iv^	a	H9-C76	2.602
	b	H16-Cl82	3.103
	c	O39-H78	2.822
	d	O39-H79	2.815
	e	H25-C86	2.786
	f	O32-H89	2.776
	g	O32-H92	2.650
	h	O70-H92	2.582
	i	H68-Cl95	3.009
TCS^...^CHT^v^	a	H63-Cl95	3.037
	b	N64-H92	2.447
	c	H68-C88	2.593
	d	O70-H79	3.074
	e	O32-H78	2.529

**Table 2:** Electronic interaction energies (ΔE_Bind_) at 0 K, enthalpy (Δ_r_H), and Gibbs energy (Δ_r_G) at 298 K for the studied complexes.

Complex	ΔE_Bind_	Δ_r_H	ΔrG
	Kcal mol^−1^		
TCS^...^CHT^i^	−10.53	−10.34	4.57
TCS^...^CHT^ii^	−4.14	−3.64	7.91
TCS^...^CHT^iii^	−17.34	−16.90	−3.14
TCS^...^CHT^iv^	−17.74	−17.13	−2.82
TCS^...^CHT^v^	−8.98	−8.14	4.05

**Table 3:** Topological parameters for the interaction between triclosan and chitosan generated in QTAIM (values in au).

Complex	Interaction *	BCP	Atoms	ρ(r)	∇^2^ρ(r)	V(r)	G(r)	H(r)
TCS^...^CHT^i^	a	BCP112	H56-Cl95	0.003407	0.011821	−0.0014	0.002177	0.000778
	b	BCP108	H38-Cl95	0.005489	0.02048	−0.00265	0.003884	0.001235
	c	BCP62	O53-H89	0.00967	0.02982	−0.0067	0.00709	0.00037
	d	BCP82	H51-C73	0.007889	0.024889	−0.00378	0.005002	0.00122
	e	BCP89	H52-C76	0.005083	0.016512	−0.0025	0.003313	0.000814
TCS^...^CHT^ii^	a	BCP19	H13-Cl81	0.00099	0.00414	−0.0004	0.00069	0.00034
	b	BCP85	N14-H80	0.0091	0.03177	−0.0054	0.00667	0.00127
	c	BCP18	H15-Cl82	0.00297	0.01164	−0.0014	0.00217	0.00074
TCS^...^CHT^iii^	a	BCP67	O58-H94	0.030714	0.090788	−0.02275	0.022722	−2.4 × 10^−5^
	b	BCP97	H63-O83	0.002765	0.011629	−0.00156	0.002233	0.000675
	c	BCP101	H68-C86	0.004168	0.012692	−0.00184	0.002508	0.000665
	d	BCP93	H44-C85	0.008847	0.030247	−0.00482	0.006191	0.001372
	e	BCP106	H25-C87	0.005063	0.017049	−0.0023	0.003281	0.000982
	f	BCP107	H25-Cl95	0.003848	0.012747	−0.00171	0.00245	0.000737
	g	BCP108	O39-H91	0.008517	0.02795	−0.00566	0.006322	0.000666
TCS^...^CHT^iv^	a	BCP81	H9-C76	0.010118	0.032934	−0.00573	0.006982	0.001251
	b	BCP87	H16-Cl82	0.003878	0.015306	−0.00193	0.002879	0.000947
	c	BCP84	O39-H78	0.0052	0.021225	−0.00306	0.004185	0.001121
	d	BCP83	O39-H79	0.005058	0.021137	−0.00304	0.004162	0.001122
	e	BCP100	H25-C86	0.006356	0.020801	−0.003	0.004101	0.001099
	f	BCP105	O32-H89	0.005646	0.022975	−0.00358	0.00466	0.001085
	g	BCP106	O32-H92	0.007102	0.026929	−0.00461	0.005673	0.001059
	h	BCP109	O70-H92	0.008081	0.025208	−0.00526	0.005782	0.00052
	i	BCP112	H68-Cl95	0.005689	0.018968	−0.00253	0.003635	0.001108
TCS^...^CHT^v^	a	BCP79	H63-Cl95	0.005274	0.016765	−0.00231	0.003252	0.000939
	b	BCP81	N64-H92	0.013362	0.032676	−0.00831	0.008237	−6.9 × 10^−5^
	c	BCP100	H68-C88	0.009361	0.029729	−0.00494	0.006186	0.001246
	d	BCP83	O70-H79	0.002613	0.010696	−0.00121	0.001943	0.000731
	e	BCP82	O32-H78	0.009564	0.03537	−0.00653	0.007684	0.001159

* Interactions highlighted in Figure 4.

## Data Availability

The authors state that data will be made available upon request.
